# Esophageal cancer with right vertebral artery variation observed during thoracoscopic esophagectomy: a case report

**DOI:** 10.1186/s12893-021-01216-0

**Published:** 2021-04-27

**Authors:** Yuta Sato, Yoshihiro Tanaka, Takeharu Imai, Yuji Hatanaka, Naoki Okumura, Nobuhisa Matsuhashi, Takao Takahashi, Kazuhiro Yoshida

**Affiliations:** Department of Surgical Oncology, Gifu Graduate School of Medicine, 1-1 Yanagido, Gifu City, 501-1194 Japan

**Keywords:** Vertebral artery, Recurrent laryngeal nerve, Thoracoscopic esophagectomy

## Abstract

**Background:**

Variation of the vertebral artery bifurcation is rare. This branching abnormality can cause unexpected vertebral artery damage and bleeding during thoracoscopic esophagectomy. There are few reports of abnormal branching of the vertebral artery associated with neurosurgery but none related to esophagectomy. We report the case together with the results of the evaluation of vertebral artery bifurcation and length in 50 patients with esophageal cancer in our hospital.

**Case presentation:**

Thoracoscopic esophagectomy was performed on a 70-year-old patient with esophageal cancer. During lymph node dissection around the right reccurent laryngeal nerve, an unusual blood vessel was found running along the right subclavian artery. We determined this blood vessel to be the right vertebral artery branching far more centrally than usual. Because this anatomical abnormality was clarified, we could then recognize that the right reccurent laryngeal nerve coursed around the right vertebral artery and the right subclavian artery and thus was running in a larger arch than usual.

**Conclusion:**

Long right vertebral artery may appear in the surgical field of the thoracoscopic esophagectomy. Knowledge of such anatomical variation is important to prevent iatrogenic injury of the right vertebral artery and the right reccurent laryngeal nerve.

## Background

The vertebral artery usually takes its origin from the subclavian artery and ascends to enter the transverse foramen of the sixth cervical vertebra [1]. There are some reports on variations of vertebral artery bifurcation [2–6], but none as they relate to esophagectomy. Knowledge of such anatomical variation is important in thoracoscopic esophagectomy to prevent iatrogenic injury of the right vertebral artery (RVA) and the right reccurent laryngeal nerve (RLN).

## Case presentation

**Fig. 1:**
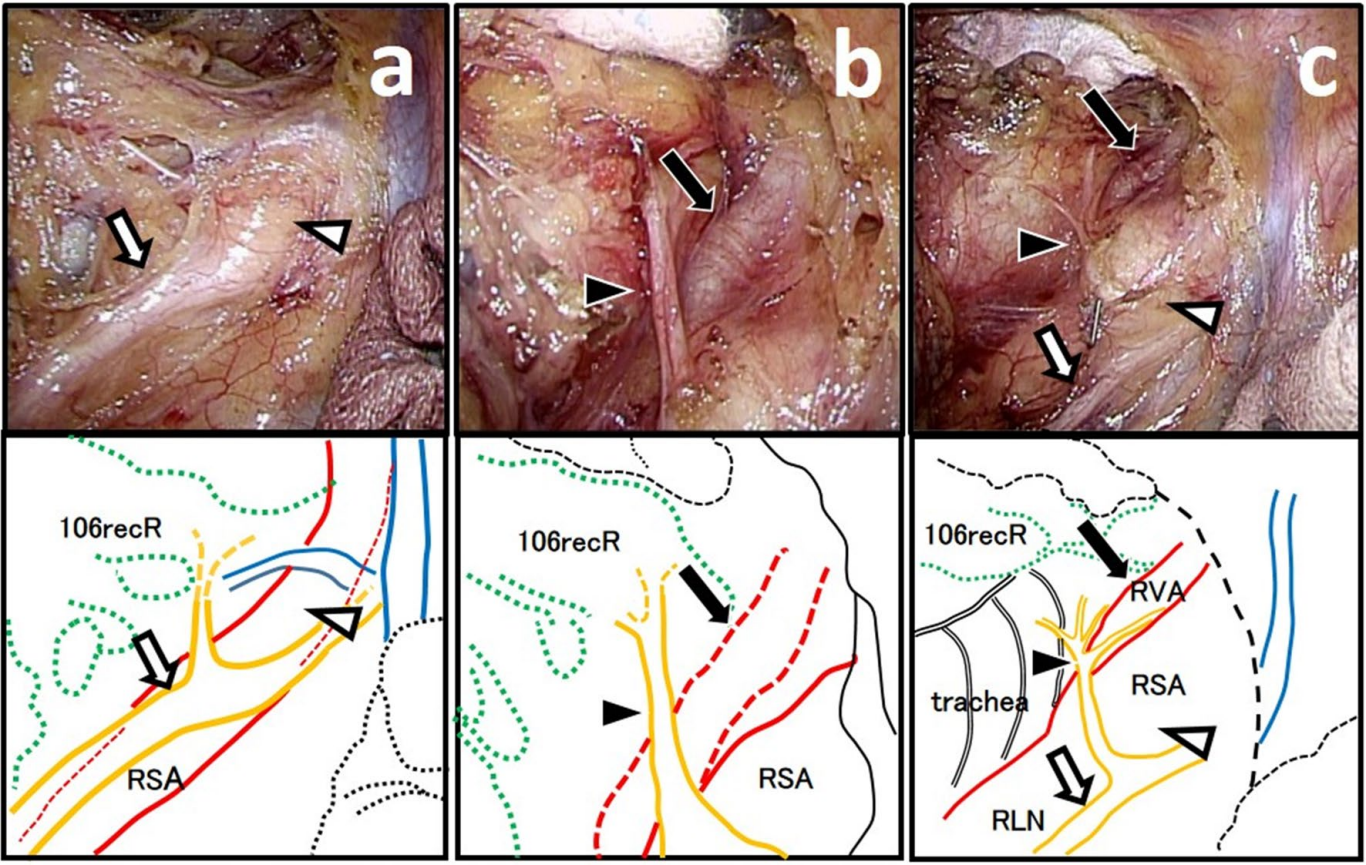
An unusual blood vessel running along the right subclavian artery was found during a thoracoscopic esophagectomy. **a** As with normal anatomy, the right vagus nerve (white arrow) running on the dorsal side of the right subclavian artery (white arrowhead). **b** An unusual blood vessel (black arrow) was found on the ventral side of the right recurrent laryngeal nerve (black arrowhead). **c** We determined this unusual blood vessel (black arrow) to be the right vertebral artery. Show the surgical field after dissection was completed

A 70-year-old man with a height of 161.0 cm, weight of 54.6 kg, and body mass index of 21.0 kg/m^2^ complained of difficulty in swallowing a meal and was referred from another hospital. He was diagnosed as having thoracic esophageal cancer (T3N1M0, Stage IIIA). We performed thoracoscopic esophagectomy in the prone position, two-fields lymphnode dissection, subtotal gastric tube reconstruction via the posterior mediastinal route, and jejunostomy after 2 courses of chemotherapy (bi-weekly DCF: docetaxel 35 mg/m^2^, cisplatin 40 mg/m^2^, fluorouracil 400 mg/m^2^) [7]. The thoracoscopic operation time was 299 min, and blood loss was 20 ml. During lymph node dissection around the right RLN, an unusual blood vessel was found running along the right subclavian artery (RSA). We assumed that the blood vessel was the RVA, which was very long at 7.02 cm (Fig. 1). Because this anatomical abnormality was clarified, we could recognize that the right RLN coursed around the RVA and the RSA and thus was running in a larger arch than usual. As a result, lymph node dissection could be performed safely without damaging the RVA and the right RLN, and no metastasis was found in the excised station number of 106recR.

## Discussion and conclusions

The RSA branches from the brachiocephalic artery, runs laterally across the anterior and middle oblique muscles, and bifurcates into the RVA as its first branch. The RVA often enters the transverse process of the sixth cervical vertebra after bifurcation. In the presented case, the RVA branched from the RSA at a very central location, so it appeared in the surgical field during lymph node dissection around the right RLN as an unusual blood vessel running along the RSA.

**Fig. 2:**
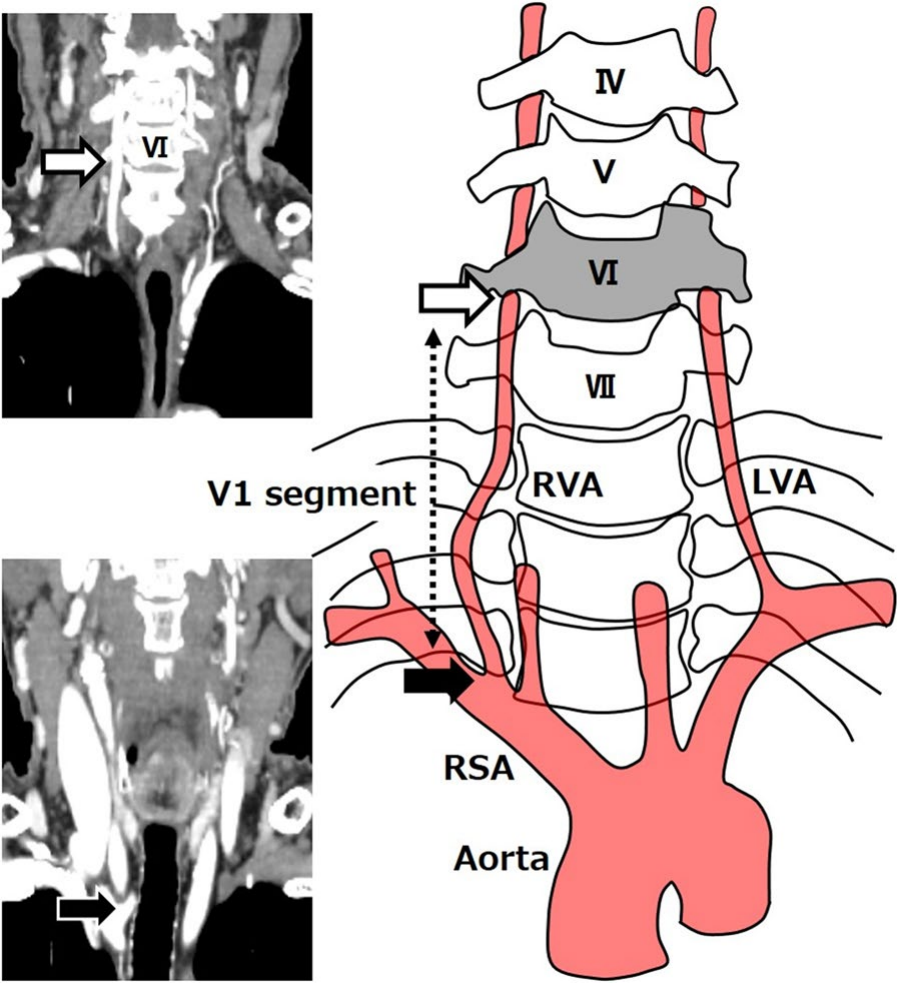
Preoperative CT image of the patient presented in our case. The RVA from the bifurcation of the SRA (black arrow) to the transverse process of the sixth vertebral body (white arrow) is called segment V1. In the presented case, V1 oh the RVA was 7.02 cm

In the vertebral artery, the segment from the bifurcation of the RSA to the transverse process of the vertebral body is called the V1 segment [8]. In a study of 266 cases, Woraputtaporn et al. reported that the average length of the RVA and left vertebral artery (LVA) V1 segments was 3.88 ± 1.14 cm [9]. The average length of the RVA was 3.69 cm and that of the LVA was 4.14 cm for 50 esophageal cancer patients operated on at our hospital. This is almost the same as those reported by Woraputtaporn et al. but V1 of the RVA of our presented case was 7.02 cm (Fig. 2), which was very long.

**Fig. 3:**
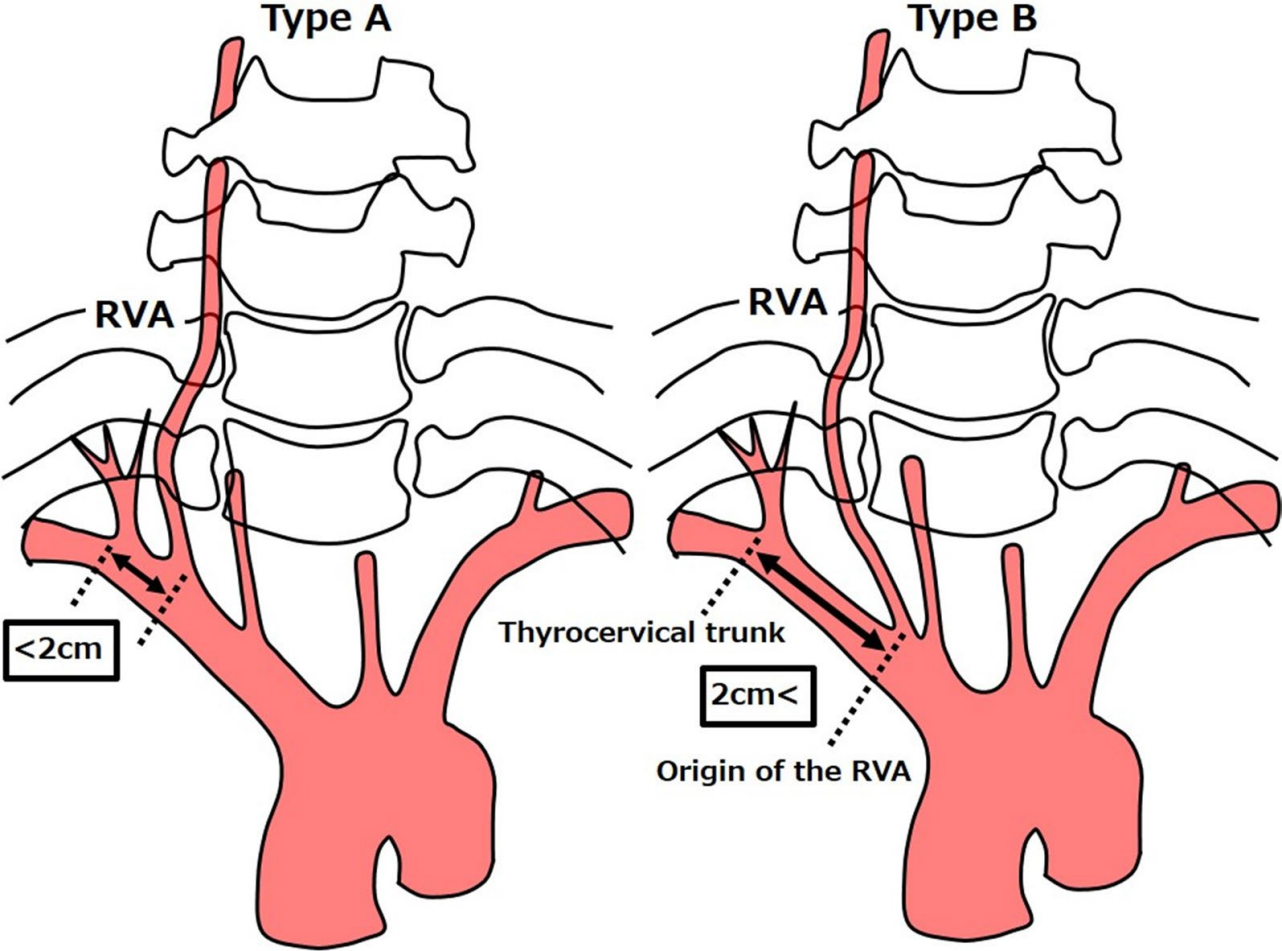
In Type A, the RVA branches within 2 cm from the thyrocervical trunk, whereas in Type B, the RVA branches more than 2 cm away from the thyrocervical trunk

Several variations have been reported at the origin of the RVA, and Newton and Potts classified them [10]. Type A, which branches within 2 cm from the thyrocervical trunk, is reported to be the most common at 83.12%, and Type B, which branches more than 2 cm away from the thyrocervical trunk, is much less common at 8.27% (Fig. 3). In the 50 patients with esophageal cancer in our hospital, Type A was seen in 45 patients (90%) and Type B, including that in the presented case, was seen in 5 patients (10%). The length of the V1 segment was compared between Type A and B. The median RVA length of Type B was 5.05 cm, which was significantly longer than 3.56 cm measured in the RVA length of Type A (*p* = 0.0020), although there were no significant differences in patients’ height between Type A and Type B (Table 1).

**Table 1:** Median length of vertebral artery, height and weight of 50 patients in our hospital

	Type A	Type B	*P* Value
Number of cases	n = 45	n = 5	
Length of RVA (cm)	3.56 [1.87–5.24]	5.05 [4.12–7.02]	0.0020
Length of LVA (cm)	3.96 [2.05–8.89]	4.47 [3.78–5.08]	0.1501
Height (cm)	163.0 [147.1–178]	168.3 [161.4–170.1]	0.2014
Weight (kg)	51.8 [36.7–89.1]	57.0 [40.1–65.9]	0.3483

The Mann–Whitney test was used for continuous variables. A *p* value of less than 0.05 was considered to be statistically significant

*RVA* right vertebral artery, *LVA* left vertebral artery

We considered that the RVA length of Type B might be longer than Type A because the RVA branched from the central side of the RSA in Type B. So, the RVA of Type B may be visible like our case in the surgical field, we will see the right RLN as if which was ascending across the two subclavian arteries, during lymph node dissection around the right RLN in thoracoscopic esophagectomy. In this type, the right RLN coursed around the RVA and the RSA and thus was running in a larger arch than usual. Therefore, preoperative understanding of RVA variation can prevent unexpected injury of the RVA and the right RLN and provide safe lymph node dissection around the right RLN in thoracoscopic esophagectomy.

## Data Availability

Not applicable.

## Abbreviations

RVA: Right vertebral artery
RLN: Recurrent laryngeal nerve
RSA: Right subclavian artery
LVA: Left vertebral artery
CT: Computed tomography

## References

[1] Maiti TK, Konar SK, Bir S, Nanda A, Cuellar H (2016). Anomalous origin of the right vertebral artery: incidence and significance. World Neurosurg.

[2] Akdeniz B, Yilmaz E, Pekel N, Ergul BU (2007). Anomalous origin of the right vertebral artery from the ascending aorta in the presence of an aberrant right subclavian artery. Int J Cardiovasc Imaging.

[3] Poynter C (1916). Arterial anomalies pertaining to the aortic arches and the branches arising from them. Nebr Univ Stud.

[4] Wasserman BA, Mikulis DJ, Manzione JV (1992). Origin of the right vertebral artery from the left side of the aortic arch proximal to the origin of the left subclavian artery. AJNR Am J Neuroradiol.

[5] Albayram S, Gailloud P, Wasserman BA (2002). Bilateral arch origin of the vertebral arteries. AJNR Am J Neuroradiol.

[6] Argenson C, Francke J, Sylla S, Dintimille H, Papasian S, diMarino V (1980). The vertebral arteries (segments V1 and V2). Anat Clin.

[7] Tanaka Y, Yoshida K, Yamada A, Tanahashi T, Okumura N, Matsuhashi N (2016). Phase II trial of biweekly docetaxel, cisplatin, and 5-fluorouracil chemotherapy for advanced esophageal squamous cell carcinoma. Cancer Chemother Pharmacol.

[8] Bruneau M, Cornelius JF, Marneffe V, Triffaux M, George B (2006). Anatomical variation of the V2 segment of the vertebral artery. Oper Neurosurg.

[9] Woraputtaporn W, Ananteerakul T, Iamsaard S, Namking M (2019). Incidence of vertebral artery of aortic arch origin, its level of entry into transverse foramen, length, diameter and clinical significance. Ant Sci Int.

[10] Newton TH, Potts DG (1974). Radiology of the skull and brain ANGIOGRAPHY vol2 arteries.

